# Fatty Acids: Essential Nutrients and Important Biomarkers

**DOI:** 10.3390/biom12091250

**Published:** 2022-09-07

**Authors:** Michail I. Gladyshev

**Affiliations:** 1 Institute of Biophysics, Krasnoyarsk Scientific Center, Siberian Branch, Russian Academy of Sciences, 660036 Krasnoyarsk, Russia; glad@ibp.ru; 2 Institute of Biophysics SB RAS, Siberian Federal University, 660041 Krasnoyarsk, Russia

Fatty acids (FA) are well-known, important components of human nutrition. Among FAs, two long-chain polyunsaturated acids of the omega-3 family—eicosapentaenoic acid (20:5n-3, EPA) and docosahexaenoic acid (22:6n-3, DHA)—play essential roles for the healthy functioning of the cardiovascular and neural systems. Since humans primarily obtain EPA and DHA from foods of aquatic origin, which are inadequate sources, a number of challenges face environmental and food sciences. These challenges are described in [1], which was published in the first Special Issue of *Biomolecules*, entitled “Fatty Acids in Natural Ecosystems and Human Nutrition”. Besides their essential roles in human health, FAs are commonly used as important biomarkers, which allow trophic webs to be traced in natural ecosystems and enable the label information of fish products on shelves to be verified. The second Special Issue of Biomolecules, entitled “Fatty Acids in Natural Ecosystems and Human Nutrition 2021” (https://www.mdpi.com/journal/biomolecules/special_issues/Fatty_Acids_2021, accessed on 30 August 2022), describes important updated findings related to FAs as essential nutrients and ecological biomarkers. 

Gladyshev et al. [2] studied the FAs available in the muscle tissue of six species and forms of charr of the genus *Salvelinus* from lakes and aquaculture. Two species, *S. boganidae* and *S. drjagini*, had the highest EPA and DHA contents in their biomass and thereby could be recommended as promising species for aquaculture, with especially high nutritional value. Moreover, the authors recommended a number of fatty acids as biomarkers for differentiating between wild and farmed charr, enabling the trade label information of fish products to be verified.

Murzina et al. [3] explored the effects of laser irradiation on lipids and fatty acids, as well as on the energy metabolism enzymes of underyearlings of wild Atlantic salmon. Increased weight gain and lower morality rates among the underyearlings in the irradiated group were found; additionally, the changes in the interrelated indicators of lipid metabolism were described. 

Tonning et al. [4] evaluated the role of lipids in the adaptation of the young-of-the-year striped bass (*Morone saxatilis*) to winter stresses. These fish were not originally utilizing energy reserves in previously described ways, and appeared to rely on other lipid classes more than on triacylglycerol and phosphatidylethanolamine. 

Merdzhanova et al. [5] studied EPA and DHA contents in several food species of fish, mollusks, and shrimps from the Black Sea. In the study, the amount of seafood necessary to provide the minimum recommended intake of omega-3 polyunsaturated fatty acids was quantified.

Godínez-Méndez et al. [6] explored α-galacto-oligosaccharides (GOS) of white lupin *Lupinus albus* as prebiotics in promoting the growth of mouse gut microbiota, with a metabolic output of short chain fatty acids (SCFAs). The authors highlighted the health benefits of lupin-GOS administration on the preservation of the intestinal microbiota and the promotion of SCFA production.

Prokopkin et al. [7] compared the quantitative fatty acid signature analysis (QFASA) method and the isotope-mixing model IsoError method—based on the compound-specific isotope analysis of fatty acids (CSIA-FA)—to determine the diets of aquatic animals. The QFASA model is a more reliable method for determining the contribution of different food sources to the diet of the experimental species, *Daphnia*, than the CSIA-based mixing model.

Strandberg et al. [8] experimentally studied the combined effects of dietary polyunsaturated fatty acids (PUFA) and higher water temperatures on the proportions of EPA and arachidonic acid (20:4n-6, ARA) in larvae of *Chironomus riparius*. They found that water warming may have cascading effects throughout the prevalence of PUFA-poor cyanobacteria as food items and a concomitant decrease in EPA in *C. riparius* as fish food. 

Voronin et al. [9] explored lipids and fatty acids in muscles of beaked redfish, *Sebastes mentella*, in a depth gradient. At all depths, high contents of DHA and EPA were observed in the redfish muscles, which was supposed to be the species’ trait. In turn, dietary markers of zooplankton (copepods), 20:1n-9 and 22:1n-11, were found to have lower contents in fish sampled from greater depths.

Finally, Taipale et al. [10], studying the relict amphipod *Pallaseopsis quadrispinosa*, explored the influence of starvation and subsequent feeding (feed used: algae with different nutritional qualities) on FA retention, compound-specific isotopic carbon fractionation, and biosynthesis of n-3 and n-6 PUFA. This experimental study revealed that FA composition of algae, namely greens vs. diatoms, had a great impact on FA biosynthesis, retention, and turnover. 
